# Altered Skeletal Muscle Phosphate Metabolism After Muscle Damage Is Associated With Elevated Effort Perception During Exercise in Humans

**DOI:** 10.1111/sms.70352

**Published:** 2026-07-29

**Authors:** Jamie Stewart McPhee, Aneurin James Kennerley, Jean‐Christophe Lagacé, James McStravick, Susan Pinner, Fabio Zambolin

**Affiliations:** ^1^ School of Sport and Exercise Sciences Manchester Metropolitan University Manchester UK; ^2^ Wolfson ACTIVE Laboratory Manchester Metropolitan University Institute of Sport Manchester Metropolitan University Manchester UK; ^3^ Faculty of Physical Activity Sciences Université de Sherbrooke Sherbrooke Quebec Canada; ^4^ Research Centre on Aging, Sherbrooke Geriatric Institute Université de Sherbrooke Sherbrooke Quebec Canada

**Keywords:** exercise‐induced muscle damage, inorganic phosphate, magnetic resonance spectroscopy, muscle metabolism, perceived exertion

## Abstract

It remains unclear why exercising damaged muscles is perceived as disproportionately more effortful. Given the known role of inorganic phosphate (Pi) in fatigue and afferent sensory signaling, we tested whether muscle damage disrupts phosphate metabolism and whether this is associated with heightened ratings of perceived exertion (RPE). Eighteen adults (23 ± 4 years) completed assessments before and 48 h after muscle damage induced by unilateral eccentric knee extensions (EIMD); the contralateral leg served as control. Magnetic resonance imaging quantified quadriceps cross‐sectional area (Qcsa) and ^31^P‐MR spectroscopy quantified Pi, phosphocreatine (PCr), Pi/PCr, ATPγ and pH at rest, during knee‐extension exercise, and recovery. RPE was recorded throughout exercise. At 48 h, the EIMD leg showed increased soreness (17‐fold), reduced maximum voluntary contraction (−18.4% ± 3.8%) and increased Qcsa (+2.8% ± 0.4%; mean individual percentage changes ± SEM; *p* < 0.001). Visit × condition interactions were observed for Pi, Pi/PCr, and ATPγ at rest (ηp^2^ = 0.31–0.35) and across rest, exercise and recovery (ηp^2^ = 0.66–0.78), but not for PCr or pH. This reflected sustained elevations of Pi and Pi/PCr, and lower ATPγ in the EIMD leg alongside higher RPE at 48 h. Elevated resting Pi/PCr was strongly associated with elevated RPE (*r* = 0.856, *p* < 0.001), whereas exercise‐induced Pi/PCr changes were not associated with RPE. Overall, EIMD causes a sustained disturbance in muscle phosphate homeostasis that is strongly associated with elevated RPE. These findings provide new evidence linking resting muscle phosphate‐metabolic disturbance with heightened effort perception after damage.

## 1 Introduction

Inorganic phosphate (Pi) plays a key role in total cellular energy balance alongside adenosine triphosphate (ATP) and phosphocreatine (PCr). In healthy skeletal muscle, Pi concentrations are tightly regulated, primarily through the activity of membrane sodium–phosphate co‐transporters [1]. However, elevated Pi levels in human muscles have been reported across a range of clinical conditions including nerve damage [2, 3], myotonic dystrophy [4, 5], inclusion body myositis [6, 7], Duchenne muscular dystrophy [8, 9], muscles lacking utrophin [10], fibromyalgia syndrome [11, 12], and peripheral vascular disease [13]. These conditions have been associated with underlying inflammation, muscle weakness [14], post‐exercise malaise, and increased perception of effort during physical activity [15, 16, 17, 18]. Similar elevations in Pi and increases in perceived effort are also observed following limb immobilization [19] and exercise‐induced muscle damage [20]. Together, these observations suggest a potential link between Pi accumulation and effort perception; however, the physiological mechanisms connecting these phenomena remain unclear.

Subjective perceptions of effort during exercise, also known as ratings of perceived exertion (RPE), are a function of the complex interactions of sensory peripheral and central inputs to the brain. While the precise mechanisms remain unclear, even for healthy individuals [21], one possibility is that central processes transfer motor cortex commands to the sensory cortex, and the RPE is scaled proportionately [22]. Another possibility is that afferent nerve activity from active skeletal muscles and other peripheral systems also contribute to the RPE [23]. Hence, disruptions in the sarcolemma membranes, the extra cellular matrix, energy metabolism and/or localized inflammation observed in the previously mentioned conditions may lead to sensitized skeletal muscle nerve afferents and, ultimately, to heightened RPE [24, 25, 26, 27]. Interestingly, exercise‐induced muscle damage (EIMD) mimics these disruptions [28] and provides a model to study the mechanisms of afferent sensitisation and changes in muscle Pi, alongside their relationship with RPE, without the confounding effects of comorbidities or pharmacological interventions.

Muscle damage can be induced through high intensity eccentric exercise, where muscles are repeatedly lengthened under tension [28]. EIMD is characterized by muscle weakness and delayed onset of muscle soreness lasting several days [28]. Interestingly, the EIMD experimental model also causes Pi to increase for several days [29, 30, 31, 32, 33] and affected individuals also experience heightened RPE during exercise [34, 35, 36]. The causes of heightened RPE remain unclear. In previous studies, the increased RPE corresponded with increased cardiorespiratory function [34, 35, 36]. However, several other studies reported a disproportionate increase in RPE beyond changes to heart rate, minute ventilation, oxygen uptake or respiratory exchange ratio [34, 37, 38, 39, 40].

The increased RPE beyond changes to cardiorespiratory function could reflect central processes, whereby greater motor commands are required to produce the same absolute external force in damaged muscles. Because EIMD reduces maximal force capacity, performing the task at the same load represents a higher relative intensity. If perceptions of effort reflect the magnitude of central motor command, then the increase in RPE may be expected to broadly track the reduction in maximal voluntary contraction compared with baseline. Alternatively, the increased RPE might be linked with heightened muscle afferent activity arising from changes to muscle mechanical and metabolic environments of damaged muscle. Muscle nerve afferents respond to mechanical and metabolic stimuli and relay this information to the central nervous system, including the sensory cortex [41, 42]. Structural and metabolic disturbance, including membrane damage and immune cell infiltrations to damaged muscles [43, 44, 45], could therefore increase afferent signaling and contribute to the heightened RPE during exercise. One metabolic candidate with a known role in muscle fatigue is Pi [46, 47] and there is evidence that Pi stimulates muscle nerve afferents directly [48], or may represent wider metabolic or structural changes that stimulate afferents.

Until now, the link between elevated Pi, reduced muscle function and increased RPE has not been described. Therefore, the aim of this study was to examine possible associations between phosphorus metabolism, muscle function and RPE after EIMD. We conducted a contralateral‐leg controlled experiment to test the hypothesis that the increase of Pi in damaged quadriceps would be associated with muscle weakness and heightened RPE during unilateral isometric knee extension exercise. This exercise was sustained for 3 min against a constant external load during which neuromuscular fatigue develops, requiring increasingly greater voluntary effort to sustain the same absolute force. Muscular phosphorus metabolism was probed using non‐invasive ^31^P magnetic resonance spectroscopy (MRS) during both rest and the isometric exercise.

## 2 Methods

### 2.1 Participants

Participants (*n* = 18, mean age = 23 ± 4 years; 11:7 male:female) provided written, informed consent to take part in the study. The study received ethical approval from the Faculty of Science and Engineering Research Ethics and Governance Committee (reference number: 37464) and conformed to the Declaration of Helsinki except for registration in a database. The inclusion criteria were males or females aged 18–30 years and willing to abstain from caffeine and large meal consumption for 2 h prior to participation, as well as alcohol usage and intense exercise for 2 days prior to any testing session. Exclusion criteria included: use of non‐steroidal anti‐inflammatory medication (NSAIDS), contraindications for MRI, presence of injury or medical conditions that prevented resistance exercise participation, regularly engaged in competitive sports or resistance training.

### 2.2 Experimental Design

Participants visited the Wolfson ACTIVE laboratory at Manchester Metropolitan University for a familiarization session, verbal explanation of the study procedures and to agree an appointment for the first (baseline) experimental session and the follow‐up which took place 48 h after baseline (48 h EIMD). Assessments at baseline and 48 h EIMD followed the same procedures, with the exception that the EIMD exercise protocol was completed only once (on the dominant leg), which was at the end of the baseline session.

### 2.3 Questionnaires and Soreness Assessment

A physical activity readiness questionnaire PAR‐Q was completed and standing stature to the nearest 0.1 cm and body mass to the nearest 0.1 kg were measured using calibrated scales (Seca GmbH, Germany) with participants wearing light clothing and barefoot. Participants were not required to be fasted. Perceived muscle soreness of the knee extensor muscles was measured using a visual analogue scale (VAS_SQ_) as participants held a single‐leg squat with the knee flexed at approximately 90° [34]. The assessment was made by asking participants to mark an “X” along a 10 cm scale to indicate the level of soreness. Anchors for the scale were “no muscle soreness” at 0 cm and “muscles are too sore to continue” at 10 cm [34, 38].

### 2.4 Structural MR Imaging

We used an in‐house Siemens MAGNETOM Vida (Siemens Healthcare GmbH, Erlangen, Germany) 3 Tesla MRI system (running XA30 software) for imaging/spectroscopy assessments of the thigh muscles. Subjects were positioned feet‐first supine (legs fully extended), and all MRI sequences were centred in line with the superior border and at the widest part of the thigh/quadriceps (occurring 3–5 cm proximal to the mid‐femur). Participants were provided with suitable hearing protection. The total scan time, including setup, was approximately 60 min [49]. An integrated radiofrequency (RF) body coil was used for ^1^H signal transmission. RF reception used both an integrated spine coil (within the participant bed) and a 16‐channel ^1^H flex coil covering the anterior surface of both thighs for structural imaging of the quadriceps. T1‐weighted images (TR 800 ms, TE 12 ms) were acquired across 28 slices (0.5 × 0.5 × 7 mm voxels) and used to assess muscle cross sectional area.

### 2.5 ^31^P‐MR Spectroscopy Assessment

**FIGURE 1:**
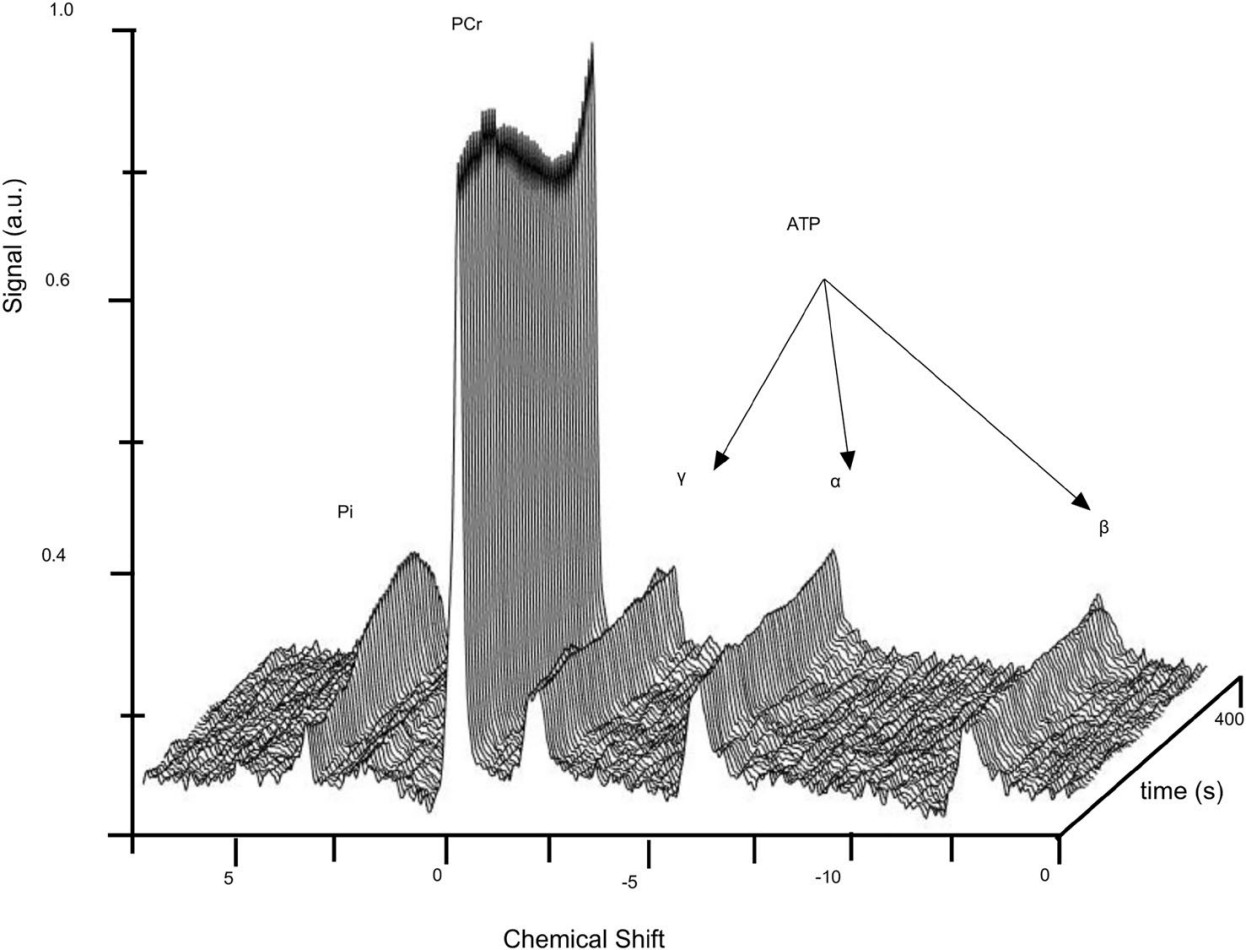
Representative ^31^P spectroscopy trace of skeletal muscle phosphate peaks. The data are from a single individual showing sequential measurements throughout rest, knee extension exercise, and recovery.

Phosphorus metabolism was probed using a dual‐tuned single channel ^1^H/^31^P surface coil (RAPID Biomedical GmbH, Ref #01150) placed on the anterior thigh of the dominant leg. The knee was raised to rest on a firm bolster 14.5 cm high (approximately 20° and the leg centred to the bore, thus placing the RF coil at the MRI isocentre to maximize the signal‐to‐noise ratio) [49]. Spectral acquisitions (TR = 550 ms, TE = 0.23 ms; Bandwidth = 2000 Hz (equivalent to 40 ppm spectral width); 1024 points over 512 ms; 90° flip angle; two step phase cycling; 10 averages (with 4 preparation scans); total duration ~7.7 s) were collected sequentially across a 6 min period to capture rest (~1 min), submaximal isometric exercise (~3 min), and recovery phases (~2 min). A representative ^31^P spectroscopy trace is shown in Figure 1.

Submaximal unilateral isometric knee extensor exercise involved raising the lower leg as the knee rests on the 14.5 cm firm bolster, with a 3 kg sandbag strapped firmly around the distal tibia close to the malleoli. This action relies on sustained contraction of the quadriceps muscles, and the 3 kg load is equivalent to approximately 10%–15% of MVC at extended knee angles based on reference values [50] and the measured MVC (Table 1). This load was selected for participants to complete the exercise task at moderate intensity without reaching task failure or incurring motion artifacts in the scanner. Previous studies showed that typical young adults can sustain isometric contractions at 20% MVC for 4–5 min [51, 52, 53].

**TABLE 1:** Skeletal muscle and resting phosphorus metabolite responses to EIMD.

	EIMD leg		Control leg		Condition × visit (*p*‐values)
	Pre	48 h post	Pre	48 h post	
MVC (kg)	64.7 ± 23.4	51.4 ± 16.4^a^	61.9 ± 21.2	62.3 ± 22.4	< 0.001
Qcsa (cm^2^)	78.9 ± 19.5	81.2 ± 20.4^a^	77.7 ± 17.3	77.3 ± 17.0	< 0.001
VAS_SQ_ (mm)	2.3 ± 1.5	39.5 ± 27.3	1.7 ± 1.5	1.7 ± 1.3	< 0.001
Resting PCr (A.U ×10^4^)	94.6 ± 15.9	92.5 ± 16.7	95.1 ± 15.1	94.2 ± 15.2	0.446
Resting Pi (A.U ×10^4^)	11.8 ± 2.5	15.6 ± 4.5^a^	12.0 ± 2.4	11.7 ± 1.9	0.002
Resting Pi/PCr (%)	12.4 ± 2.6	16.7 ± 3.8^a^	12.6 ± 2.4	12.4 ± 1.6	< 0.001
ATPγ (A.U ×10^4^)	25.6 ± 4.6	22.9 ± 4.5^a^	25.9 ± 4.9	26.2 ± 4.3	< 0.001
pH	7.11 ± 0.02	7.11 ± 0.02	7.11 ± 0.02	7.11 ± 0.02	0.182

*Note:* The values are presented as group means ± SD. Percentage changes reported in the text are calculated as mean individual within‐participant changes from baseline. Measurements were obtained at baseline and 48 h following eccentric exercise in the EIMD leg and contralateral control leg. The condition × visit *p*‐values represent the interaction effects from repeated measures ANOVA.

Abbreviations: ATP, adenosine tri phosphate; MVC, maximum voluntary contraction; PCr, phosphocreatine; Pi, inorganic phosphate; Qcsa, peak value for quadriceps anatomical cross‐sectional area; VAS_SQ_, Visual Analog Scale for Soreness.

^a^ Indicates significantly different from baseline values within the same leg (*p* < 0.05).

Rate of perceived exertion was assessed on the 6–20 Borg scale [54] at the end of every minute during the 3 min isometric sustained contraction. RPE was defined as a conscious sensation of how hard, heavy and strenuous the task is, to depend mainly on the sense of effort and the exercise‐evoked somatic sensation [55]. Measurements were taken at baseline and 48 h EIMD. Measurements were also repeated on the non‐dominant leg, which acted as a control.

### 2.6 Maximal Voluntary Contraction Assessment

Participants sat upright on a custom‐made knee extension dynamometer with hips and knees flexed at 90° and straps secured around the waist to minimize extraneous movements. Single leg maximal voluntary knee extension isometric contraction (MVC) was tested with the leg secured 2 cm proximal to the malleolus by an inextensible strap connected at the other end to a calibrated load cell. Force signals were amplified and recorded (PowerLab 16/30; ML880, ADInstruments, Bellavista, NSW, Australia), with real‐time visual display of force available on a computer monitor. A warm‐up was provided consisting of six isometric contractions lasting approximately 3 s at 50%–80% maximal effort. After a 1 min rest, participants performed 3 isometric MVCs with each lasting approximately 3 s and separated by 1 min rest. The highest external force value was accepted as participant's MVC [56]. This procedure was then repeated for the other leg.

### 2.7 Exercise Induced Muscle Damage Protocol

The EIMD protocol consisted of eccentric knee extensor contractions of the dominant leg (EIMD leg), while the non‐dominant leg remained rested and acted as control. A Kineo Multistation machine (Globus, Italy) was used, providing isokinetic mode and enabling the eccentric load to be accurately and rapidly adjusted in relation to the concentric load. A warm‐up was provided consisting of 10 isokinetic concentric leg extensions performed through the full range of motion, with each lasting approximately 3 s. The maximal concentric and eccentric torques were assessed separately. The concentric torque started with a flexed knee and worked through the full range of motion at 60°/s. The eccentric torque started with an extended knee and worked through the full range of motion at 60°/s. Participants completed repeated sets of 10 dynamic eccentric knee extensions in isotonic mode, with the load set at 100% of the eccentric peak torque, and participants were asked to give a maximal effort to oppose the load. To return to the starting position for each eccentric contraction, participants produced a moderate intensity concentric contraction at 50% of concentric peak torque. At the end of each set of 10 repetitions, an MVC isometric force was tested, and exercise was terminated when MVC was reduced by 40% compared with starting values [57, 58].

### 2.8 Data Handling and Statistical Analysis

MRI data were acquired and processed using the Syngo.Via software (VB60S_HF03, Siemens Healthcare GmbH, Erlangen, Germany). DICOM images of the thigh transverse sections were analyzed by manually tracing the quadriceps cross sections using Osirix software (v12.5.2) and the largest cross‐sectional area was accepted as Qcsa. Intramuscular pH was calculated using the chemical shift of the Pi resonance in relation to the PCr peak, using the Henderson‐Hasselbalch equation [59]. Absolute metabolite quantification was not performed, so phosphorus peaks are reported in arbitrary units (AU). To minimize between‐session variability, the same MRI system, RF coil, acquisition parameters, participant positioning procedures, anatomical localisation, and post‐processing pipelines were used across all sessions. The controlled repeated‐measures design allowed comparisons to be performed within participants, with the contralateral leg serving as a control. Because EIMD may alter individual phosphorus metabolites differently, Pi, PCr, and ATPγ peaks are reported separately, while the Pi/PCr ratio was included as an internally normalized measure that is less sensitive to global variation in signal amplitude than individual metabolite peaks.

Paired *t*‐tests were performed at baseline to compare resting values for control and EIMD legs within participants. The presence of muscle damage was assessed using two‐way repeated measures ANOVA with within‐subject factors for visit (baseline and 48 h) and condition (control and EIMD) for MVC, Qcsa, and VAS_SQ_.

Resting metabolite levels were analyzed using two‐way repeated measures ANOVA (visit × condition) to evaluate changes in resting muscle Pi, PCr, Pi/PCr, ATPγ, and pH values.

To evaluate metabolite responses during the MRI exercise protocol, continuous metabolite data were summarized into three predefined workload phases: Rest (before commencement of exercise), Exercise (onset to completion of the 3 min contraction), and Recovery (post‐exercise). For each participant, we calculated mean values for visit, condition, and workload phases for Pi, PCr, Pi/PCr, ATPγ, and pH. Phase‐averaged values were analyzed using three‐way repeated‐measures ANOVA with within‐subject factors for visit, condition and workload phase. Type III sums of squares were used and where sphericity assumptions were violated, Greenhouse–Geisser corrections were applied. When significant visit × condition, or visit × condition × workload phase interactions were detected, post hoc comparisons were used with estimated marginal means and Bonferroni‐adjusted contrasts to test whether the change from baseline to 48 h differed between conditions (EIMD and control legs) within each workload phase.

**FIGURE 2:**
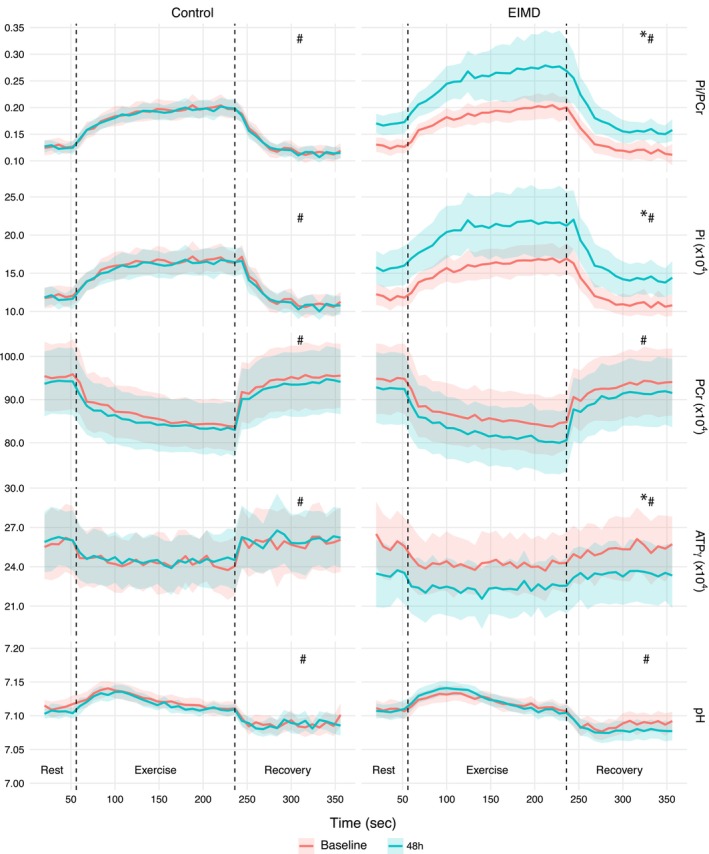
Skeletal muscle phosphate responses across rest, exercise, and recovery before and after exercise‐induced muscle damage. Sequential measurements were taken throughout rest, knee extension exercise, and recovery at baseline and 48 h following muscle‐damaging eccentric exercise for the Control leg (left panels) and the EIMD leg (right panels). Metabolites include inorganic phosphate (Pi), phosphocreatine (PCr), Pi/PCr ratio, ATPγ, and pH. Data are presented as mean ± 95% confidence intervals. Gray shaded regions indicate the period of sustained isometric knee‐extensor exercise performed inside the MRI scanner. # indicates significant workload effect; * indicates significant condition × visit interaction (*p* < 0.05).

To characterize dynamic changes in phosphorus metabolites during exercise onset and early recovery, linear slopes were fitted to the first 60 s of exercise and the first 30 s of recovery (where the changes were approximately linear for Pi/PCr, refer to Figure 2) for each participant, visit, and condition. Slopes were compared between visits and conditions using linear mixed‐effects models with participant as a random effect. Additional regression analyses examined whether between‐visit slope changes (48 h − baseline) differed by condition while adjusting for baseline slope.

Associations between markers of muscle damage and changes in phosphorus metabolites were assessed using Pearson's product–moment correlations. For between‐visit analyses, change values were expressed as fold‐changes calculated from the 48 h value divided by the baseline value (where a value of 1 indicates no change, above 1 indicates an increase and below indicates a decrease from baseline). Where indicated, partial correlations were used to adjust for covariates, including MVC fold‐change. Stepwise multiple linear regression was used to identify independent associations with RPE fold‐change, entering candidate markers including MVC, Qcsa, and resting fold‐changes in Pi/PCr, ATPγ, and pH. Additional regression analyses evaluated whether exercise‐induced metabolite changes were associated with RPE change during exercise (calculated using the averages of rest values and the final 32 s exercise).

All statistical analyses were performed in RStudio (version 2024.04.2 + 764) running R (version 4.4.1; R Foundation for Statistical Computing, Vienna, Austria) using “tidyverse” for data management, “afex” for repeated‐measures ANOVA, “emmeans” for post hoc contrasts, “effectsize” for partial eta squared (ηp^2^), and “MASS” for stepwise regression. Statistical significance was set at two‐tailed *α* < 0.05. Effect sizes were calculated using Cohen's d for *t*‐tests and partial eta squared (ηp^2^) for repeated‐measures ANOVA, interpreted using standard thresholds (small = 0.01, medium = 0.06, large = 0.14).

## 3 Results

### 3.1 Participants and Response to Eccentric Exercise

Eighteen participants were included (11 men and 7 women, mean ± SD age: 23.1 ± 3.9 years; height: 171.6 ± 11.0 cm; body mass: 69.9 ± 13.0 kg; and BMI: 23.6 ± 2.4 kg/m^2^). A further two volunteers were excluded after presenting with MRI contraindications.

At baseline, there were no significant differences between the left and right legs for MVC, Qcsa or the resting muscle phosphorus measurements (all *p* > 0.05; Table 1). EIMD was present in the eccentrically exercised leg (EIMD) at 48 h post exercise with significant condition × visit interactions for MVC (ηp^2^ = 0.30) and Qcsa (ηp^2^ = 0.30) (both *p* < 0.001; Table 1). At 48 h, the mean (± SEM) individual percentage change from baseline for the EIMD leg was 18.4% ± 3.8% reduction of MVC and 2.8% ± 0.4% increase of Qcsa (both *p* < 0.001). There was also a 17‐fold increase of soreness indicated from the VAS_SQ_ (baseline: 2.3 ± 1.5 mm, 48 h: 39.5 ± 27.3 mm; Cohen's *d* = 1.92; both mean ± SD). In contrast, the control leg showed no significant changes from baseline for all measurements (Table 1).

Significant visit × condition interactions were found for resting Pi (*p* = 0.002, ηp^2^ = 0.31), Pi/PCr (*p* < 0.001, ηp^2^ = 0.35) and ATPγ (*p* < 0.001, ηp^2^ = 0.34), whereas PCr (*p* = 0.446), and pH (*p* = 0.182) showed no significant interactions (Table 1).

### 3.2 Impact of EIMD on Phosphorus Metabolite Responses to Exercise

There were significant differences across rest, exercise and recovery workload phases for all metabolites: Pi/PCr (ηp^2^ = 0.93), Pi (ηp^2^ = 0.91), PCr (ηp^2^ = 0.80), ATPγ (ηp^2^ = 0.73), and pH (ηp^2^ = 0.86) (all *p* < 0.001, Figure 2). Pi and Pi/PCr increased from rest to exercise and returned toward baseline during recovery. PCr and ATPγ both decreased during exercise and recovered thereafter, while pH was lowest during recovery. There were no significant visit (baseline vs. 48 h) × workload phase interactions for Pi, PCr, ATPγ, or pH (all *p* > 0.05).

There were significant visit × condition interactions for Pi (ηp^2^ = 0.71) and Pi/PCr (ηp^2^ = 0.78) (both *p* < 0.001). There was also a moderate, significant three‐way interaction for Pi/PCr (visit × condition × workload phase; *p* = 0.037, ηp^2^ = 0.20), as the response to exercise was greater at 48 h in the EIMD leg. Post hoc comparisons demonstrated that Pi and Pi/PCr were elevated in the EIMD leg at 48 h during rest, exercise, and recovery (all *p* < 0.001). Analysis of the exercise‐onset kinetics showed that the rise in Pi/PCr during the first 60 s was 54% steeper in the EIMD leg at 48 h, but this did not reach statistical significance (*p* = 0.056). Early recovery kinetics did not differ between visits (*p* = 0.498).

PCr showed a strong significant effect of workload (*p* < 0.001, ηp^2^ = 0.80) and a moderate effect of visit (*p* = 0.025, ηp^2^ = 0.29), but no significant visit × condition interaction (*p* = 0.810, ηp^2^ = 0.004). Post hoc analyses confirmed no differential 48 h effect between conditions across any phase.

ATPγ showed strong significant effects of workload (*p* < 0.001, ηp^2^ = 0.73), visit (*p* = 0.012, ηp^2^ = 0.35), and condition (*p* = 0.001, ηp^2^ = 0.52). ATPγ differed between conditions at 48 h, as shown by the significant visit × condition interaction (*p* < 0.001, ηp^2^ = 0.66) and post hoc contrasts confirming lower ATPγ in the EIMD leg at 48 h during rest, exercise, and recovery (all *p* ≤ 0.005). This effect was consistent across workload phases (no significant three‐way interaction, *p* = 0.230).

There was a strong effect of workload phase on pH (*p* < 0.001, ηp^2^ = 0.86), with lower values during recovery compared with rest and exercise. There were no significant main effects of visit or condition, and no significant visit × condition interaction.

No significant condition effects were observed on the exercise‐ and recovery‐kinetics slopes of PCr, ATPγ, or pH responses to workload transitions (all *p* ≥ 0.05). The phosphorous changes for transitions from rest to exercise were not significantly correlated with MVC, either at baseline or at 48 h (all *p* > 0.05).

### 3.3 Rating of Perceived Exertion During Exercise

**FIGURE 3:**
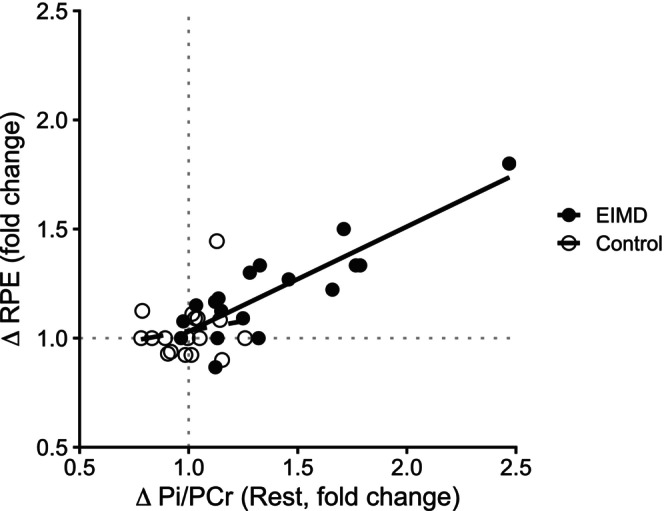
Associations between fold‐changes in Pi/PCr and rating of perceived exertion in Control and EIMD legs. Filled circles and solid trend line represent the EIMD leg and open circles with dashed line represent the Control leg. Light gray dotted horizontal and vertical lines represent a fold‐change value of 1, indicating no change from baseline.

The RPE measured at the end of 3 min isometric contraction showed a significant condition × visit interaction (*p* = 0.002; ηp^2^ = 0.48), as RPE increased in the EIMD leg at 48 h compared with baseline (mean ± SD: 14.3 ± 2.1 vs. 12.0 ± 2.2, respectively) but remained unchanged in the control leg (mean ± SD: 12.5 ± 2.1 vs. 12.3 ± 2.2 for 48 h and baseline, respectively). The fold‐change in RPE was strongly correlated with the fold‐changes in MVC (*r* = −0.755, *p* < 0.001; 95% CI: −0.90 to −0.44), Qcsa (*r* = 0.642, *p* = 0.004; 95% CI: 0.23 to 0.86), and resting Pi/PCr (*r* = 0.856, *p* < 0.001; 95% CI: 0.65 to 0.95; Figure 3). No significant correlations were observed in the control leg (all *p* ≥ 0.05; Table 2). After controlling for the fold‐change in MVC using partial correlation, the association between fold‐changes in RPE and Pi/PCr in the EIMD leg remained significant (partial *r* = 0.720, *p* = 0.001; 95% CI: 0.36 to 0.89), whereas no significant association was observed in the control leg (partial *r* = 0.230, *p* = 0.399; CI: −0.29 to 0.65).

**TABLE 2:** Bivariate relationships between fold‐change in markers of muscle damage, perceived exertion, and resting phosphorus metabolite values.

		EIMD leg			Control leg		
		∆MVC	∆Qcsa	∆RPE	∆MVC	∆Qcsa	∆RPE
**∆Pi**	*r*= *p*=	**−0.631** **0.005**	**0.562** **0.015**	**0.832** **< 0.001**	−0.117 0.656	−0.011 0.966	0.169 0.518
**∆PCr**	*r*= *p*=	0.371 0.129	−0.383 0.117	−0.233 0.351	0.209 0.421	−0.128 0.625	−0.063 0.810
**∆Pi/PCr**	*r*= *p*=	**−0.675** **0.002**	**0.598** **0.009**	**0.856** **< 0.001**	−0.168 0.519	0.023 0.930	0.185 0.477
**∆pH**	*r*= *p*=	0.127 0.616	0.210 0.402	**−0.502** **0.034**	0.037 0.887	0.365 0.150	−0.159 0.543
**∆ATPγ**	*r*= *p*=	**0.723** **0.001**	**−0.531** **0.023**	**−0.594** **0.009**	−0.027 0.917	0.197 0.450	−0.130 0.620

*Note:* Values represent Pearson correlation coefficients (*r*) and corresponding *p*‐values describing associations between the fold‐changes from baseline (∆) in muscle function, morphology, and perceived exertion with fold‐changes in resting phosphorus metabolite levels between baseline and 48 h. Correlations are presented separately for the EIMD leg and the control leg. Bold values indicate statistically significant relationships.

Abbreviations: ∆ATPγ, fold‐change in γ‐adenosine triphosphate; ∆MVC, fold‐change in maximal voluntary contraction; ∆PCr, fold‐change in phosphocreatine; ∆pH, fold‐change in intracellular pH; ∆Pi, fold‐change in inorganic phosphate; ∆Pi/PCr, fold‐change in inorganic phosphate to phosphocreatine ratio; ∆Qcsa, fold‐change in quadriceps anatomical cross‐sectional area; ∆RPE, fold‐change in rating of perceived exertion.

Stepwise regression was performed with fold‐change in RPE as the dependent variable and MVC, Qcsa, Pi/PCr (rest), ATPγ (rest), and pH (rest) fold‐changes as the candidate predictors. For the EIMD leg, the final model identified only resting fold‐change in Pi/PCr (*p* = 0.023) as independently associated with fold‐change in RPE (adjusted *R* ^2^ = 0.716, *p* < 0.001). In the control leg, none of these factors were significantly associated with fold‐change in RPE (full model adjusted *R* ^2^ = 0.294, *p* = 0.505).

There was no correlation between the RPE reported after 3 min exercise and the Pi/PCr at the end of 3 min exercise, either at baseline (EIMD leg: *r* = 0.189; *p* = 0.454, 95% CI: −0.31 to 0.60; control leg: *r* = 0.295; *p* = 0.251, 95% CI: −0.22 to 0.68) or at 48 h (EIMD leg: *r* = 0.435; *p* = 0.072, 95% CI: −0.04 to 0.75; control leg: *r* = −0.185; *p* = 0.478, 95% CI: −0.61 to 0.32). Stepwise regression taking fold‐change in RPE as the dependent variable and entering exercise‐induced fold‐changes in Pi/PCr, ATPγ and pH revealed that none were associated with fold‐change in RPE (adjusted *R* ^2^ = −0.194, *p* = 0.970).

## 4 Discussion

### 4.1 Summary of Main Findings

The principal novel finding of this study was a large elevation in Pi and Pi/PCr in the damaged muscle 48 h after eccentric exercise, which was evident across rest, exercise and recovery phases. Crucially, the increase in resting Pi/PCr was very strongly associated with an elevated perception of effort during muscle contractions, while the acute exercise‐induced changes in Pi/PCr were not associated with perceived effort. Together, these findings provide new evidence that EIMD alters the phosphate metabolic profile of skeletal muscle, and the resting metabolic disturbance is strongly associated with the elevated perception of effort when exercising damaged muscles.

### 4.2 Resting Muscle Pi/PCr Is Associated With RPE When Exercising Damaged Muscles

Skeletal muscles are prone to damage during unaccustomed eccentric exercise [57] and to this end we observed 18% lower MVC at 48 h post EIMD, which was similar to past studies [58, 60, 61]. The observed increase in Qcsa indicated localized swelling [62, 63], and muscle soreness [24, 64]. The resting muscle showed a 33% increase in the Pi/PCr ratio, which was primarily due to the elevated Pi rather than changes in PCr levels. Several previous studies have reported similar elevations of Pi/PCr for resting muscles after EIMD [3, 29, 30, 31, 32, 33]. There are also similar reports in skeletal muscles after limb immobilization [19], nerve damage [2, 3], myotonic dystrophy [4, 5], inclusion body myositis [6, 7], Duchene muscular dystrophy [8, 9], muscles lacking utrophin [10], fibromyalgia syndrome [11, 12] and peripheral vascular disease [13] and in cardiac muscles of type 2 diabetics and patients after cardiomyopathy [65, 66, 67]. It therefore appears to signify underlying cellular damage with metabolic disruptions.

None of the previous studies that identified elevated Pi/PCr in damaged muscles or long‐term conditions specified the possible links with physical function. We show for the first time that the increase of resting Pi/PCr was inversely correlated with the change in MVC and was positively correlated with the increased Qcsa 2 days after EIMD. There was also a strong association with the increased RPE when damaged muscles were exercised, which was independent of the changes to MVC and Qcsa. To the best of our knowledge, this is the first study to link Pi accumulations of rested, damaged muscles with the increased perceptions of effort when performing moderate intensity exercise.

Evidence exists to demonstrate the involvement of muscle afferents with RPE [68], but their role is still debated [69]. Type III and IV muscle afferents transmit information to the central nervous system in response to metabolic, mechanical and other localized signals [70]. They are sensitive to changes in the muscle environment, triggering perceptions of pain, fatigue [71] and effort during exercise [69, 72]. Our results revealed differences in the relationship between the muscle metabolic environment and RPE for healthy compared with damaged muscles during the sustained moderate intensity isometric knee extensor exercise. It is possible that Pi stimulates muscle nerve afferents [48, 73], but our methodology does not allow this to be confirmed and our findings may not necessarily reflect a causal link of Pi accumulations and increased RPE. It is also possible that the raised Pi reflects other metabolite and structural changes to which muscle afferents are sensitized and can influence RPE.

The available literature raises the possibility of an alternative mechanism that excludes the role of muscle afferents in favor of a direct scaling between central motor commands (known as corollary discharge) with RPE [22, 74, 75]. Our results do not rule out this effect because a greater motor command would be necessary after EIMD to complete isometric exercise using the same absolute external load. However, this was unlikely to be the main cause of elevated RPE because the increase of resting Pi/PCr after EIMD was not related to the decrease in MVC following multiple regression analysis.

### 4.3 Transitions From Rest to Exercise and Recovery

Our results confirm that damaged muscles transitioning from rest to exercise follow similar relative changes for Pi, PCr, Pi/PCr, ATPγ, and pH compared with undamaged muscles (strong workload effects, Figure 2). However, the absolute values for Pi and Pi/PCr were consistently higher in the damaged muscles. This pattern suggests that EIMD altered the metabolic baseline of the damaged muscle, rather than changing the overall metabolic response to exercise. There was a 54% steeper rise in the slope of Pi/PCr during the onset of exercise in damaged muscles, but this did not reach the level of significance (*p* = 0.056). This suggests a possible alteration in early exercise kinetics, but the overall metabolic responses to exercise remained broadly preserved after EIMD. Consistent with this, neither the rise of Pi/PCr from rest to exercise nor the absolute Pi/PCr values at end exercise were associated with the RPE responses in healthy muscles or 2 days after EIMD. One possible explanation for the strong RPE association with resting Pi/PCr in damaged muscles, but not with exercising Pi/PCr, is that afferents were already sensitized by structural and metabolic changes as well as inflammation from infiltrating immune cells [76, 77]. Moreover, a recent study showed a close association between Pi and neuromuscular fatigue, more so than muscle acidosis [78].

### 4.4 Elevated Pi/PCr in Damaged Muscles

It remains unclear why Pi/PCr is elevated in damaged skeletal muscles at rest. In healthy muscles, Pi concentrations are tightly regulated to minimize losses to extracellular compartments which would reduce the total cellular energy potential. A net influx of Pi from extracellular to intracellular spaces while at rest might be one potential mechanism for the Pi/PCr elevation after EIMD, but we are not aware of any reports to this effect. Pi influx occurs by active transport against its electrical and concentration gradients via sodium‐phosphate cotransporters, driven by the electrochemical gradient of sodium [79, 80, 81]. Since sodium movements across the membrane are low at rest, a net Pi influx seems unlikely to explain our observations. Nevertheless, we noted an increase of Pi even when expressed as a proportion of total phosphates (i.e., Pi/(PCr + ATP)) from 0.1 in both legs at rest and in the control leg at 48 h, up to 0.14 in the EIMD leg at 48 h, which is very similar to the increase described by Aldridge et al. [32]. The occurrence of muscle damage is well known after eccentric contractions [44, 82] as sarcomeres attempting to shorten are forcibly lengthened causing high tension [28] affecting extracellular structures, the sarcolemma and myofibrils [83]. It is possible that some Pi movement could occur as membranes can become permeable, even to large enzymes such as creatine kinase [84, 85].

Another potential explanation for elevated resting Pi/PCr is that the rate of ATP hydrolysis may have increased. Possible causes of this could include the repair of cellular damage [76, 77], increased passive muscle tension caused by elevated resting sarcoplasmic Ca^2+^ [63], and increased activity of ion pumps along the sarcolemma affected by membrane damage [86]. Pi may also accumulate if oxidative metabolism in the mitochondria is impaired and slows the recovery of PCr, either due to changed mitochondrial function or to impaired microvascular function reducing the oxygen delivery [87]. Indeed, the results of Pathare et al. [19] showed that the increase of Pi (3 mM) in rested muscle after immobilization was almost equally offset by the small absolute decrease of PCr (2 mM) [19]. Several previous studies reported altered glycolytic and mitochondrial processes during incremental leg extension exercise affecting PCr recovery dynamics [88].

### 4.5 Strengths and Limitations

The present study has several strengths. The controlled unilateral design allowed each participant to serve as their own control, enabling direct comparisons between damaged and undamaged muscle within the same physiological environment. The use of in vivo ^31^P‐MRS provided non‐invasive measurement of skeletal muscle phosphate metabolism, while the integration of metabolic, functional, and perceptual outcomes during dynamic measurements inside the MRI scanner allowed a comprehensive assessment of the physiological responses to EIMD.

Some considerations should also be noted when interpreting the findings. The study involved healthy young adults who were not engaged in competitive sport, and therefore the results may not directly generalize to other populations. Measurements were obtained at a single time point (48 h) following EIMD, corresponding to the period when muscle damage symptoms are typically pronounced, but the full time course of metabolic changes during recovery was not assessed. In addition, the exercise task was performed under controlled unilateral conditions inside the MRI scanner. This approach was necessary to obtain reliable metabolic measurements and minimize confounding influences of afferents from cardio‐respiratory systems affecting RPE, which would have been difficult to avoid if whole body and higher intensity exercises were included. For this reason, the findings may not fully represent responses during more complex or whole‐body exercise tasks. Although the use of the same fixed load for every participant during exercise could theoretically produce different relative exercise intensities between individuals, this is unlikely to have substantially influenced the present findings. Ratings of perceived exertion increased consistently in the EIMD leg across participants, and there were no associations between baseline or 48 h MVC and the corresponding changes in phosphorous metabolites or RPE. These observations suggest that the relationships identified between muscle phosphate metabolism and effort perception were not driven by differences in relative workload between individuals. Resting phosphorus metabolites were reported in arbitrary units rather than absolute concentrations, as is common for non‐invasive ^31^P‐MRS studies. Careful standardization of the acquisition protocol, alongside the within‐participant design, inclusion of a contralateral control leg and use of the Pi/PCr ratio provided methodological consistency.

Future work should examine how the observed metabolic disturbances interact with neural mechanisms contributing to effort perception. This may involve the combination of ^31^P‐MRS measurements with neural assessments during exercise, such as transcranial magnetic stimulation or cortical activity measurements, to clarify how altered muscle metabolism influences central motor control following muscle damage. Further studies should also investigate the physiological mechanisms underlying the sustained disturbance in skeletal muscle phosphate homeostasis, including elevated Pi and Pi/PCr levels accompanied by reduced ATPγ concentrations at rest. We found 15% lower ATPγ levels at rest after EIMD, which is similar to the findings of Zehnder et al. [89]. Together with the increased Pi levels, these changes may influence muscle contractile function and fatigue susceptibility, as elevated Pi can impair cross‐bridge cycling [46] and alter calcium handling [47]. Future work examining the time course of these metabolic disturbances and their effects on exercise tolerance will help clarify the role of phosphate metabolism in the physiological response to muscle damage.

## 5 Conclusion

This study revealed a very strong relationship between the increased Pi/PCr of resting muscles following EIMD and the increase of RPE when exercising the same muscles. The same relationship was not evident for the changes in Pi/PCr occurring during exercise in damaged muscles, nor for Pi/PCr measured at rest or during exercise in non‐damaged muscles. These findings provide the first evidence in humans that the heightened perceptions of effort during exercise are strongly related to elevated muscle Pi/PCr in damaged muscles, independently from changes to MVC, suggesting that disturbances in phosphate metabolism may contribute to exercise intolerance after muscle damage. We highlight Pi/PCr as a potential biomarker of impaired exercise tolerance following muscle damage and provide new insights into the possible metabolic determinants of exercise intolerance in muscle disorders.

## 6 Perspectives

The approach used in the present work could be extended to better understand exercise tolerance and movement limitations in neuromuscular disorders, fatigue‐related conditions, disuse and recovery after injury or surgery where activity can feel disproportionately effortful. This work also raises the possibility that rehabilitation or intervention strategies that modify muscle damage and recovery may act partly by altering both muscle phosphate metabolism and perceived effort. A related question is whether similar metabolic and perceptual changes develop during prolonged exercise, such as marathon or ultra‐endurance events, where repeated eccentric loading, muscle damage and fatigue progressively alter how effort is perceived. Together, this work would help to determine whether altered muscle phosphate metabolism is only a marker of muscle damage, or a physiological mechanism contributing to impaired exercise tolerance that is modifiable in health, disease and athletic performance.

## Author Contributions

Jamie S McPhee: conceptualization and study design, data analysis and interpretation, manuscript writing. Jean‐Christophe Lagacé: data analysis, interpretation, reviewing the manuscript. Susan Pinner: data collection, interpretation, reviewing the manuscript. James McStravick: MRI protocol optimization, interpretation, reviewing the manuscript. Aneurin Kennerley: MRI/MRS data optimisation, data quality assurance, interpretation and manuscript review. Fabio Zambolin: conceptualization and study design, data collection, analysis and interpretation, manuscript review and approval.

## Funding

This project benefitted from funding from the European Union's Horizon 2020 research and innovation programme under the Marie Skłodowska‐Curie COFUND grant agreement No. 801604 (Doctoral Training Alliance).

## Conflicts of Interest

The authors declare no conflicts of interest.

## Data Availability

The data that support the findings of this study are available from the corresponding author upon reasonable request.
